# Treatment of allergic eosinophilic asthma through engineered IL-5-anchored chimeric antigen receptor T cells

**DOI:** 10.1038/s41421-022-00433-y

**Published:** 2022-08-16

**Authors:** Sisi Chen, Gaoying Chen, Fang Xu, Beibei Sun, Xinyi Chen, Wei Hu, Fei Li, Madiha Zahra Syeda, Haixia Chen, Youqian Wu, Peng Wu, Ruirui Jing, Xinwei Geng, Lingling Zhang, Longguang Tang, Wen Li, Zhihua Chen, Chao Zhang, Jie Sun, Wei Chen, Huahao Shen, Songmin Ying

**Affiliations:** 1 International Institutes of Medicine, the Fourth Affiliated Hospital of Zhejiang University School of Medicine, Yiwu, Zhejiang China; 2 Department of Pharmacology and Department of Respiratory and Critical Care Medicine of the Second Affiliated Hospital, Zhejiang University School of Medicine, Key Laboratory of Respiratory Disease of Zhejiang Province, Hangzhou, Zhejiang China; 3 Department of Cell Biology and Department of Cardiology of the Second Affiliated Hospital, Zhejiang University School of Medicine, Hangzhou, Zhejiang China; 4 Key Laboratory of Respiratory Disease of Zhejiang Province, Department of Respiratory and Critical Care Medicine, Second Affiliated Hospital of Zhejiang University School of Medicine, Hangzhou, Zhejiang China; 5 Department of Cell Biology and Bone Marrow Transplantation Center of the First Affiliated Hospital, Zhejiang University School of Medicine, Hangzhou, Zhejiang China; 6 Department of Anatomy, Zhejiang University School of Medicine, Hangzhou, Zhejiang China; 7 Key Laboratory for Biomedical Engineering of Ministry of Education, State Key Laboratory for Modern Optical Instrumentation, College of Biomedical Engineering and Instrument Science, Collaborative Innovation Center for Diagnosis and Treatment of Infectious Diseases, Zhejiang University, Hangzhou, Zhejiang China; 8 State Key Lab of Respiratory Disease, Guangzhou, Guangdong China

**Keywords:** Immunology, Cell biology, Biological techniques

## Abstract

Severe eosinophilic asthma (SEA) is a therapy-resistant respiratory condition with poor clinical control. Treatment efficacy and patient compliance of current therapies remain unsatisfactory. Here, inspired by the remarkable success of chimeric antigen receptor-based cellular adoptive immunotherapies demonstrated for the treatment of a variety of malignant tumors, we engineered a cytokine-anchored chimeric antigen receptor T (CCAR-T) cell system using a chimeric IL-5-CD28-CD3ζ receptor to trigger T-cell-mediated killing of eosinophils that are elevated during severe asthma attacks. IL-5-anchored CCAR-T cells exhibited selective and effective killing capacity in vitro and restricted eosinophil differentiation with apparent protection against allergic airway inflammation in two mouse models of asthma. Notably, a single dose of IL-5-anchored CCAR-T cells resulted in persistent protection against asthma-related conditions over three months, significantly exceeding the typical therapeutic window of current mAb-based treatments in the clinics. This study presents a cell-based treatment strategy for SEA and could set the stage for a new era of precision therapies against a variety of intractable allergic diseases in the future.

## Introduction

Over 339 million people suffer from asthma worldwide^1^ and patients with severe eosinophilic asthma (SEA) are at high risk of mortality and low quality of life^2–4^. SEA, characterized by eosinophilic inflammation, is a major phenotype of refractory asthma with poor clinical control^4^. Eosinophils have a prominent role in SEA pathogenesis, causing airway epithelial damage and bronchial remodeling^5^. Eosinophilia is closely related to higher exacerbation frequency and worse control, leading to decreased lung function^6,7^. Thus, strategies capable of inactivating or depleting eosinophils offer attractive therapies for SEA^8,9^.

Currently, asthma symptoms control mainly relies on the daily administration of the inhaled corticosteroids (ICS) combined with β2 receptor agonists^10^, which can cause intolerable adverse reactions, including osteoporosis and hypertension^11^. For SEA patients, biological agents targeting the interleukin-5 (IL-5)/IL-5 receptor α (IL-5Rα) axis interfere with the pathologic functions of eosinophils and show promising therapeutic effects^12^. The human IL-5Rα, showing specific binding for IL-5, is expressed on mature eosinophils, basophils, and their progenitors^13,14^. When exposed to allergens, epithelial-derived cytokine IL-33 upregulates the IL-5Rα expression on eosinophil progenitors^15^. Then the concomitant expression of IL-5 and IL-5Rα mediates the growth and terminal differentiation of eosinophil progenitors, which contributes to the subsequent development of blood and tissue eosinophilia in eosinophilic asthma patients with type 2-high inflammation^16,17^. Anti-IL-5 monoclonal antibodies, such as mepolizumab and reslizumab, have been approved for second-line treatment of SEA in the clinics by decreasing the level of eosinophils in the blood/sputum through neutralization of IL-5, and inhibition of eosinophil differentiation and activation to eventually reduce the exacerbation frequency in asthma patients^18–20^. Yet, their effects on the reduction of airway eosinophils are restricted^21,22^. Benralizumab, an mAb against the IL-5Rα, can directly eliminate eosinophils through antibody-dependent cell-mediated cytotoxicity^13^. Although benralizumab showed better efficacy than mepolizumab or reslizumab in improving pulmonary functions of SEA patients^23^, population-level response rates remain low and the reduction of annual exacerbation rates remains limited^24,25^. Moreover, poor bioavailability of mAb drugs results in a need for repeated administration over long timespans, which severely compromises patient compliance.

Recently, cellular adoptive immunotherapies based on CD19-specific CAR-T cells have shown remarkable efficacy in treating B cell malignancies^26–28^. The chimeric antigen receptor (CAR) is a fusion protein composed of an extracellular target-specific scFv-moiety and an intracellular T-cell receptor domain typically consisting of CD28 and CD3ζ, allowing for antigen-specific activation of T-cell killing in a strict scFv-dependent manner^29,30^. Using this empiric design principle, similar CAR-T strategies have been extensively tested for the treatment of other cancers such as neuroblastoma^31,32^, hepatocellular carcinoma^33–35^, as well as other diseases in the fields of autoimmune diseases^36–38^, cardiovascular diseases^39^ and senescence-associated pathologies^40^. However, neither of the latter approaches could repeat or emulate the treatment efficacy of the inaugural anti-CD19 system, which forms the basis of all clinically approved CAR-T products that have hitherto arrived onto the market^30,41–43^. In fact, a typical adverse effect of classical CAR-T therapies is the development of severe clinical anaphylaxis^44,45^. A few reports indicate that xenogenetically-derived scFv domains could largely contribute to such immunogenicity-related reactions^43,46–48^, which restricts the application of CAR-T cell therapy in allergic patients.

In this work, we developed an scFv-independent, cytokine-anchored chimeric antigen receptor (CCAR) configuration that uses IL-5 as the extracellular target-binding domain. When engineered into primary T-cells, IL-5-anchored CCAR-T cells specifically target IL-5Rα-expressing eosinophils and eosinophil progenitor cells^49,50^. IL-5-anchored CCAR-T cells effectively restricted eosinophil differentiation with obvious protection against allergic airway inflammation in murine asthma models.

## Results

### Engineering of eosinophil-targeting CCAR-T cells

**Fig. 1:**
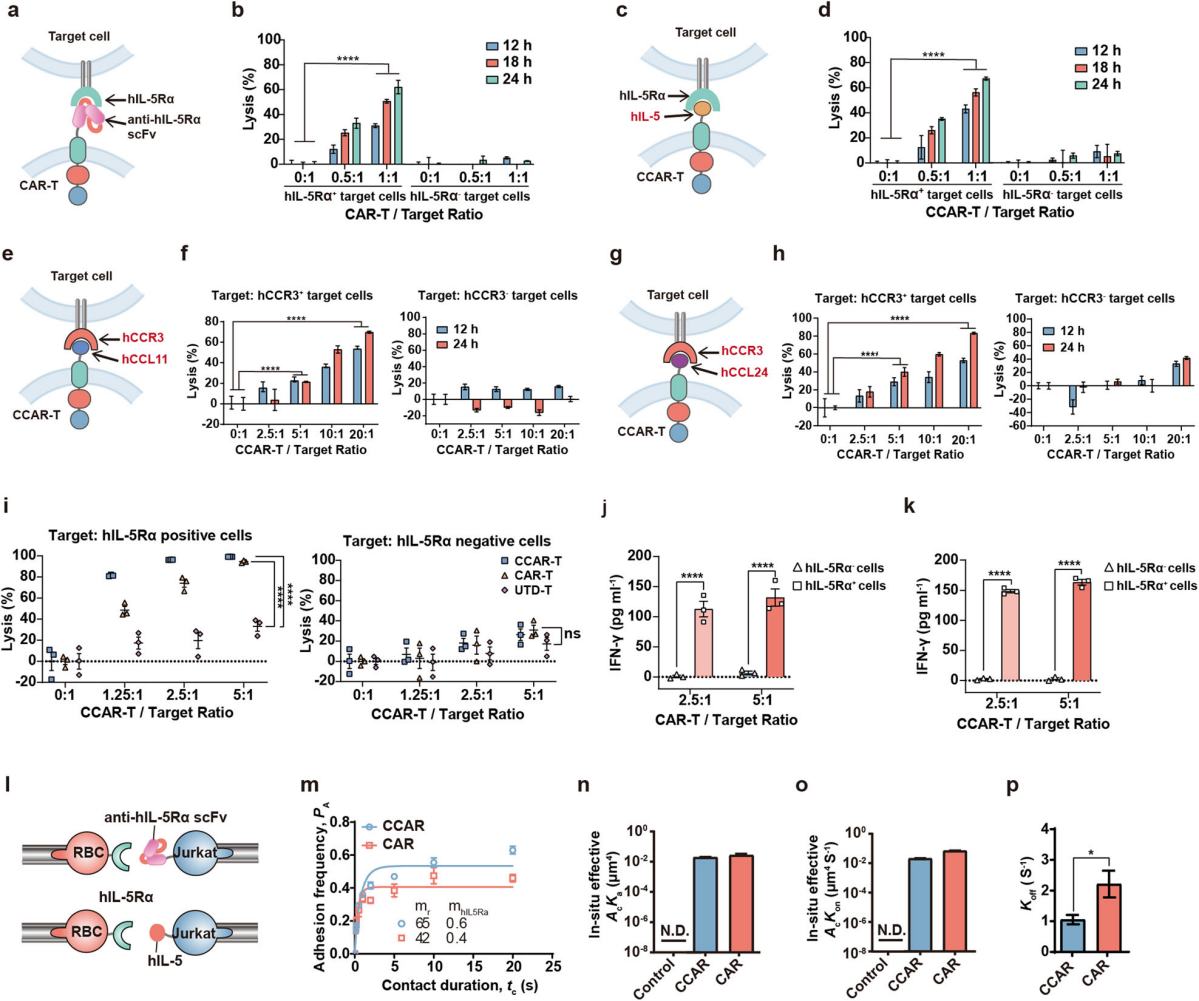
Design, characterization, and comparison of eosinophil-targeting CCAR-T cells. **a** Design of the anti-hIL-5Rα CAR-T cells using the scFv derived from human IL-5Rα mAb as the antigen-binding domain. **b** Cytotoxic activity of anti-hIL-5Rα CAR-T cells as determined by a bioluminescence assay using luciferase-expressing hIL-5Rα^+^ or hIL-5Rα^–^ U2OS cells as target cells. Differences between the CAR-T cell-treated and control group were examined by the two-way ANOVA, ^****^P < 0.0001. **c** Design of the hIL-5-anchored CCAR-T cells using human IL-5 as the antigen-binding domain. **d** Cytotoxic activity of hIL-5-anchored CCAR-T cells. Two-way ANOVA, ^****^P < 0.0001. **e** Design of the hCCL11-anchored CCAR-T cells. **f** Cytotoxic activity of the CCL11-anchored CCAR-T cells against target cells. Two-way ANOVA, ^****^P < 0.0001. **g** Design of the hCCL24-anchored CCAR-T cells. **h** Cytotoxic activity of the CCL24-anchored CCAR-T cells to target cells. Two-way ANOVA, ^****^P < 0.0001. **i** Cytotoxic activity of anti-hIL-5Rα CAR-T cells or hIL-5-anchored CCAR-T cells from healthy human donors against hIL-5Rα^+^ U2OS cells. Two-way ANOVA, ^****^P < 0.0001. UTD-T, un-transduced T cells. **j**, **k** The production of interferon-γ (IFN-γ) in the supernatant of anti-hIL-5Rα CAR-T cells (**j**) or hIL-5-anchored CCAR-T cells (**k**) from healthy human donors after coculture with target cells for 24 h was determined by ELISA kit. Two-way ANOVA, ^****^P < 0.0001. **l** Schematic diagram of the bio-membrane force probe assay for the adhesion frequency assay. **m** Binding specificities (*P*_a_) of anti-hIL-5Rα CAR/hIL-5-anchored CCAR and hIL-5Rα as measured by adhesion frequency with different contact duration. **n** In-situ effective affinity (*A*_c_*k*_a_) of the interaction between anti-hIL-5Rα CAR-hIL-5Rα or hIL-5-anchored CCAR-hIL-5Rα and hIL-5Rα. N.D., not detected. **o** In-situ effective on-rate (*A*_c_*k*_on_) of the anti-hIL-5Rα CAR-hIL-5Rα bond and the hIL-5-anchored CCAR-hIL-5Rα bond. **p** Average off-rate (*k*_off_) of the anti-hIL-5Rα CAR-hIL-5Rα bond and the hIL-5-anchored CCAR-hIL-5Rα bond. Two-tailed *t*-test, ^*^P < 0.05.

Human eosinophils and eosinophil progenitor cells highly express IL-5Rα (Supplementary Fig. S1)^49,50^, a feature that is exploited by the current benralizumab therapy^51^. To engineer eosinophil-targeting hIL-5Rα-specific T cells, we designed two different CAR configurations (Fig. 1a, c). The first design follows the conventional CAR blueprint, comprising an extracellular scFv-moiety derived from benralizumab fused to an intracellular TCR-derived CD28-CD3ζ signaling domain (anti-hIL-5Rα CAR-T; Fig. 1a). Our second design uses the human IL-5 (hIL-5) as the CAR extracellular domain instead of scFv (hIL-5-anchored CCAR; Fig. 1c). Both CAR-T variants, generated through retroviral transduction of murine primary T cells (Supplementary Fig. S2a), showed specific elimination of hIL-5Rα^+^ cells in vitro (Fig. 1b, d). C-C motif chemokine receptor 3 (CCR3) is another widely studied surface marker expressed by mature eosinophils and Th2 subsets (Supplementary Fig. S1)^52^. To engineer CCR3-specific CCAR-T cells, we designed CCL11-anchored and CCL24-anchored CCAR-T cells using human CCL11 and CCL24 as the CCR3-binding domains, respectively (Fig. 1e, g). As expected, both hCCL11-anchored and hCCL24-anchored CCAR-T cells displayed cytotoxicity against stable hCCR3-expressing target cells (Fig. 1f, h).

Next, we transduced primary T cells from healthy human donors with both lentiviral vectors (Supplementary Fig. S2b). The corresponding anti-hIL-5Rα CAR-T and hIL-5-anchored CCAR-T cells showed a similar proliferative capacity to native un-transduced T-cells (UTD-T cells) (Supplementary Fig. S3), and exhibited specific elimination of hIL-5Rα^+^ target cells as well (Fig. 1i). Furthermore, anti-hIL-5Rα CAR-T and hIL-5-anchored CCAR-T cells showed comparable IFN-γ secretion capacity after coculture with hIL-5Rα^+^ target cells for 24 h (Fig. 1j, k).

To characterize the binding affinity of hIL-5-anchored CCAR and anti-hIL-5Rα CAR to the hIL-5Rα target, we used the adhesion frequency assay as previously reported^53,54^ (Fig. 1l). Statistical analysis revealed no significant differences between these two CAR-T variants in terms of in-situ binding kinetics (Fig. 1m), i.e., effective affinity (*A*_c_*K*_a_) (Fig. 1n) and effective on-rate (*A*_c_*K*_on_) (Fig. 1o). However, the off-rate (*K*_off_) of the hIL-5-anchored CCAR was slightly lower than that of the anti-hIL-5Rα CAR (Fig. 1p).

We then transferred anti-mIL-5Rα CAR-T cells into the allergic airway inflammation model (Supplementary Fig. S4b). Anti-mIL-5Rα CAR-T cells, which showed specific elimination of mIL-5Rα^+^ cells in vitro (Supplementary Fig. S4a), could not significantly reduce eosinophil levels in vivo (Supplementary Fig. S4c, d). Meanwhile, we compared the mCCL11-anchored, mCCL24-anchored, and mIL-5-anchored CCAR-T cells in vivo (Supplementary Fig. S5a). Interestingly, neither the mCCL11-anchored nor the mCCL24-anchored CCAR-T cells reduced eosinophil levels, while mIL-5-anchored CCAR-T cells showed promising results (Supplementary Fig. S5b, c). Thus, we selected the IL-5-anchored CCAR-T strategy for further studies.

### Functional assessment of hIL-5-anchored CCAR-cells in NSG mice

**Fig. 2:**
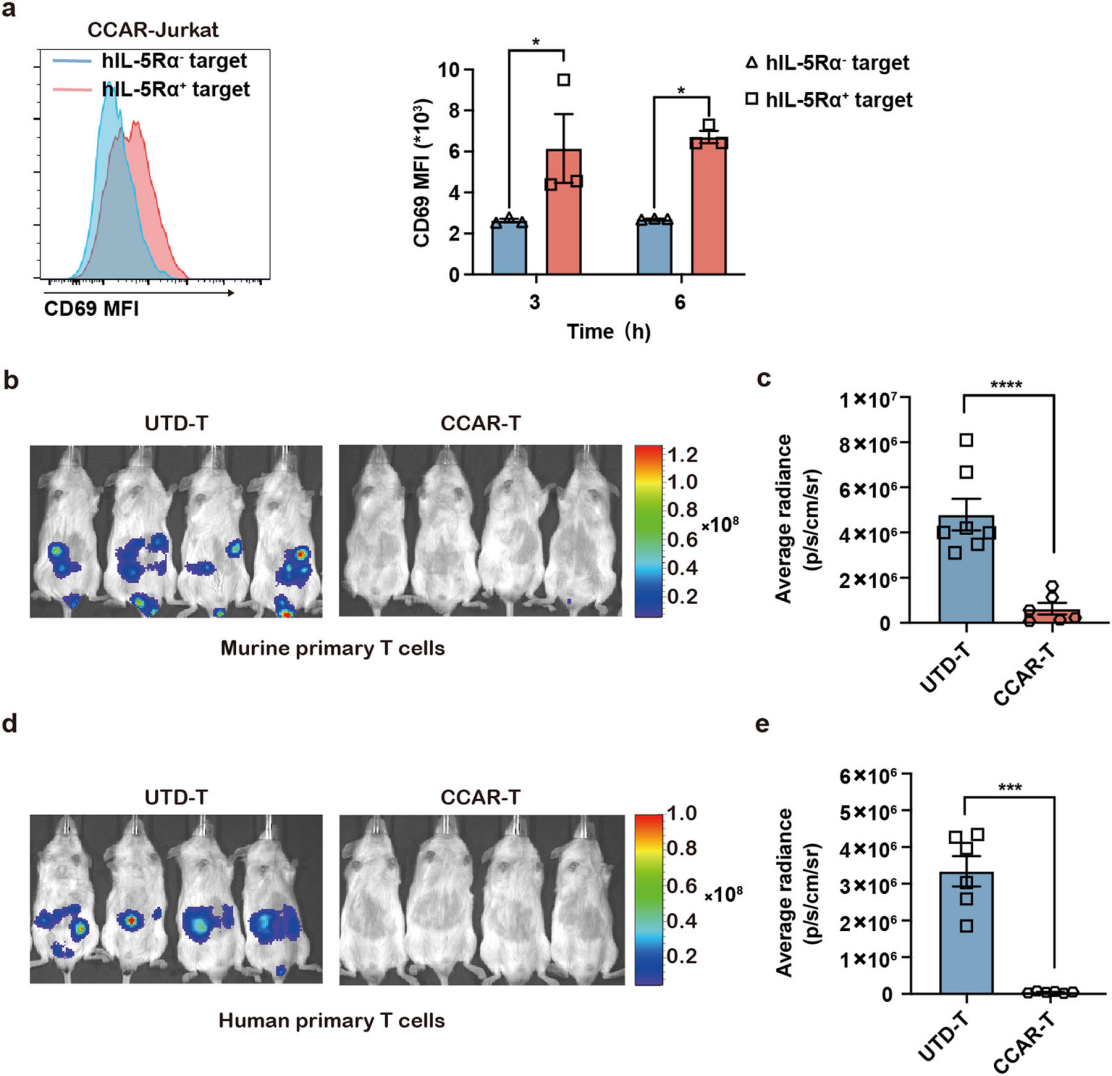
Functional assessment of hIL-5-anchored CCAR-cells in vivo. **a** flow cytometry analysis of the CD69 expression on the CCAR-Jurkat cells 3 h or 6 h after the NCG mice were injected intraperitoneally with hIL-5Rα^+^ U2OS cells and hIL-5-anchored CCAR-Jurkat cells. Two-tailed *t*-test, ^*^P < 0.05. **b** Representative bioluminescence images showing the target cell burden in the NCG mice 24 h after the hIL-5Rα^+^ U2OS cells expressing firefly luciferase and the murine primary CCAR-T cells/UTD-T cells were intraperitoneally injected. **c** The quantification of the target cell burden in mice from **b**. Two-tailed *t*-test, ^****^P < 0.0001. **d** Representative bioluminescence images showing the target cell burden in the NCG mice 24 h after the hIL-5Rα^+^ U2OS cells expressing firefly luciferase and the human primary CCAR-T cells/UTD-T cells were intraperitoneally injected. **e** The quantification of the target cell burden in mice from d. Two-tailed *t*-test, ^***^P < 0.001.

Next, we tested the function of hIL-5-anchored CCAR-T cells in vivo. First, we detected the CD69 expression on Jurkat cells to assess the CCAR-induced T cell activation. The hIL-5-anchored CCAR-Jurkat cells, which can be activated by target cell in vitro (Supplementary Fig. S6), were significantly activated by hIL-5Rα^+^ target cells as well after intraperitoneal injection of both hIL-5Rα^+^ U2OS cells and hIL-5-anchored CCAR-Jurkat cells in NOD/ShiLtJGpt-*Prkdc*^em26Cd52^*Il2rg*^em26Cd22^/Gpt (NCG) mice (Fig. 2a). Then, we tested the in vivo efficacy of hIL-5-anchored CCAR-T cells against target cells through bioluminescence imaging. Compared to UTD-T cells, both murine primary hIL-5-anchored CCAR-T cells (Fig. 2b, c) and human primary CCAR-T cells (Fig. 2d, e) dramatically eliminated hIL-5Rα^+^ U2OS cells.

### Targeting specificity of IL-5-anchored CCAR-T cells

**Fig. 3:**
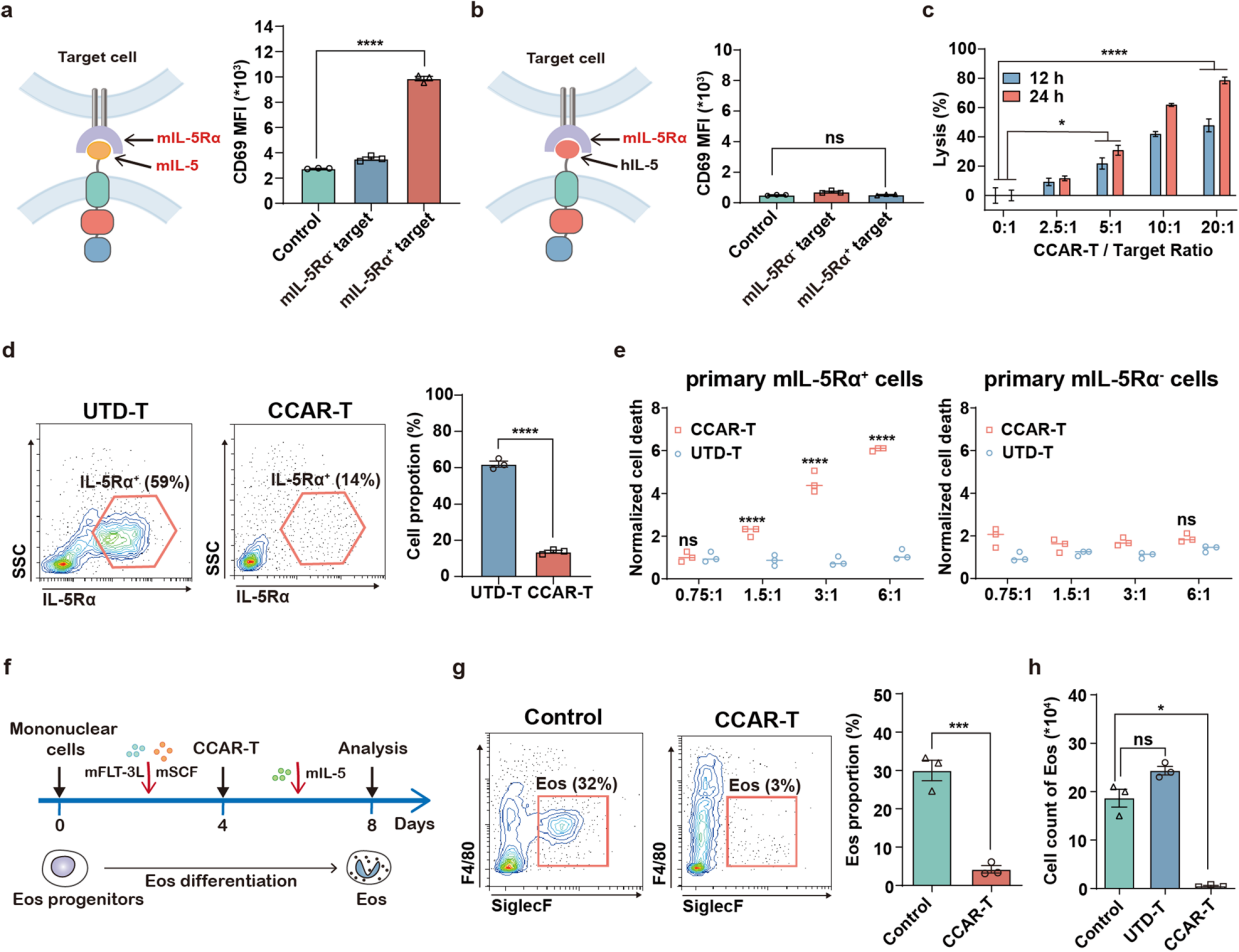
IL-5-anchored CCAR-T cells show specific cytotoxicity and inhibition of eosinophilic differentiation in vitro. **a** Jurkat cells were transduced with mIL-5-anchored CCAR comprising a mouse IL-5 linked to human CD28 costimulatory and CD3ζ signaling domains (m.IL-5-h.28z). Flow cytometry analysis of CD69 expression on mIL-5-anchored CCAR-Jurkat cells after coculture with target cells (U2OS cells) for 24 h. One-way ANOVA, ^****^P < 0.0001. **b** Jurkat cells were transduced with hIL-5-CCAR comprising a human IL-5 linked to human CD28 costimulatory and CD3ζ signaling domains (h.IL-5-h.28z). Flow cytometry analysis of CD69 expression on hIL-5-anchored CCAR-Jurkat cells after coculture with target cells for 24 h. Dunn’s Kruskal-Wallis test. **c** Primary T cells from BALB/c mice were transduced with mIL-5-CCAR comprising a mouse IL-5 linked to mouse CD28 costimulatory and CD3ζ signaling domains (m.IL-5-m.28z). Cytotoxic activity of mIL-5-anchored CCAR-T cells against mIL-5Rα^+^ U2OS cells. Two-way ANOVA, ^****^P < 0.0001, ^*^P < 0.05. **d** Flow cytometry analysis showing the proportion of primary mIL-5Rα^+^ cells derived from bone marrow after treating with mIL-5-anchored CCAR-T cells or UTD-T cells at a CCAR-T to target ratio of 6:1 in vitro for 8 h. UTD-T, un-transduced T cells. Two-tailed *t*-test, ^****^P < 0.0001. **e** Normalized cell death of primary mIL-5Rα^+^ cells or mIL-5Rα^–^ cells after treating with mIL-5-anchored CCAR-T cells or UTD-T cells. Differences between mIL-5-anchored CCAR-T cells-treated and UTD-T cells-treated group were examined by two-way ANOVA, ^****^P < 0.0001. **f** Timeline of Eos differentiation induction, mIL-5-anchored CCAR-T cell administration, and flow cytometry analysis of Eos. The mIL-5-anchored CCAR-T cells were administrated at a CCAR-T/Target ratio of 3:1. Eos, eosinophil. **g** Flow cytometry plots showing the proportion of BM-derived Eos after treating with mIL-5-anchored CCAR-T cells or not. BM, bone marrow. Two-tailed *t*-test, ^***^P < 0.001. **h** Histogram of the cell count of BM-derived Eos. Brown-Forsythe and Welch ANOVA, ^*^P < 0.05.

To assess the function of IL-5-anchored CCAR-T cells in the murine asthma models, we designed murine variants based on mIL-5-anchored CCAR-T cells or CCAR-Jurkat cells specific for murine IL-5Rα (mIL-5Rα). The mIL-5-anchored CCAR-Jurkat cells were activated by target cells expressing mIL-5Rα after 24 h of coculture (Fig. 3a), whereas hIL-5-anchored CCAR-Jurkat cells showed no response to mIL-5Rα^+^ target cells, confirming target specificity of the CCAR (Fig. 3b). Next, we transduced primary murine T cells with the mIL-5-anchored CCAR retrovirus (mIL-5-anchored CCAR-T cells) for cytotoxic activity assay. Similarly, mIL-5-anchored CCAR-T cells showed significant cytotoxicity against mIL-5Rα^+^ target cells (Fig. 3c). Further, we performed a cell apoptosis assay using mouse primary mIL-5Rα^+^ and mIL-5Rα^−^ cells as target cells. In contrast to the UTD-T cells, mIL-5-anchored CCAR-T cells remarkably reduced the proportion of mIL-5Rα^+^ cells (Fig. 3d) and displayed specific cytolysis (Fig. 3e). As the differentiation of eosinophils plays a crucial role in airway eosinophilia during SEA^16^, mIL-5-anchored CCAR-T cells were also applied on a bone marrow-derived eosinophil (BMDE) differentiation assay (Fig. 3f). We observed that administration of mIL-5-anchored CCAR-T cells could block eosinophil differentiation both on the cell proportion (Fig. 3g) and cell count level (Fig. 3h). Together, these results suggest that the mIL-5-anchored CCAR-T cells are capable of selectively and effectively eliminating eosinophils.

### Protective effect of CCAR-T cells against allergic eosinophilic inflammation

**Fig. 4:**
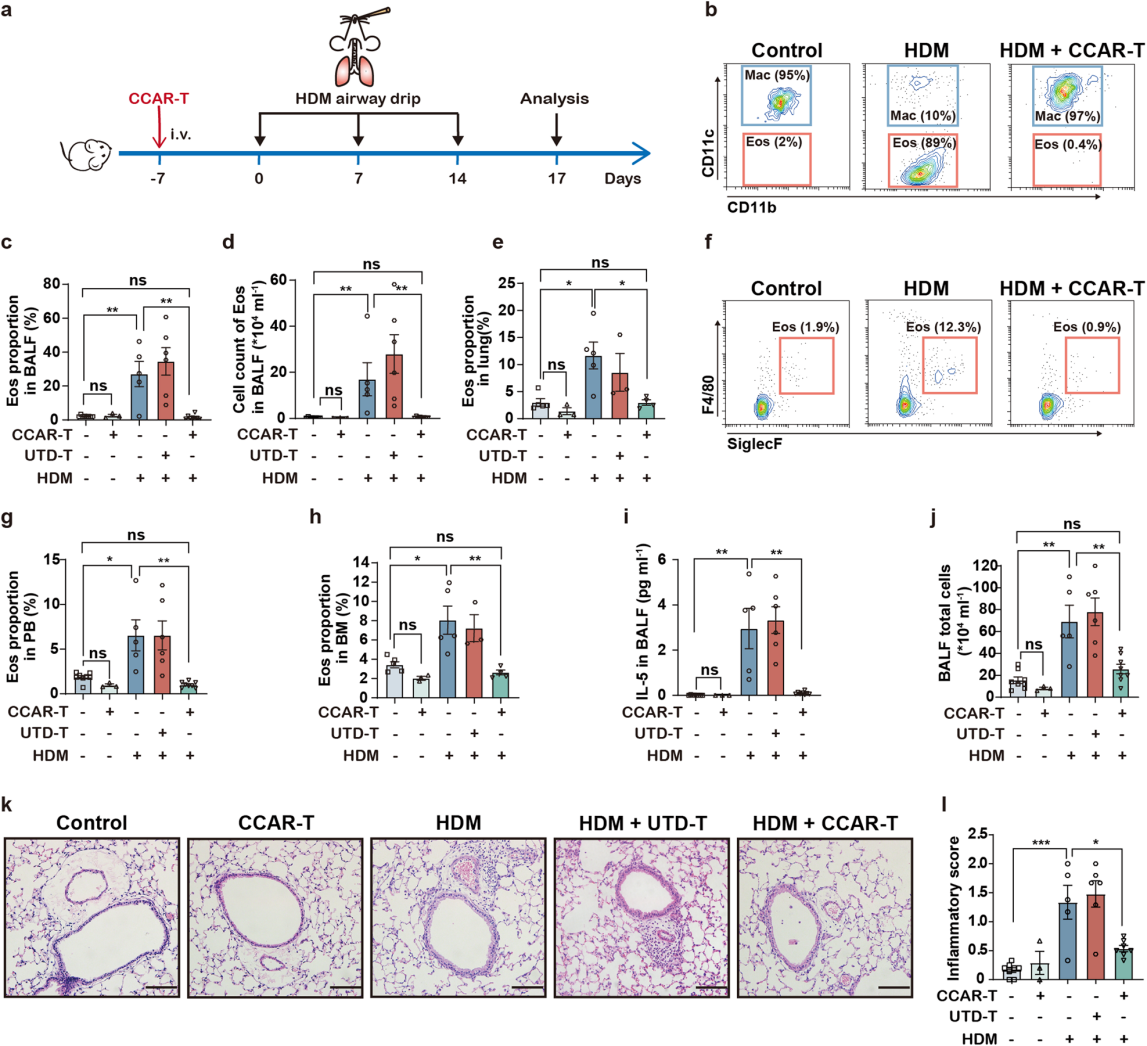
IL-5-anchored CCAR-T cells eliminate eosinophils and protect against HDM-induced allergic airway inflammation in vivo. **a** Timeline of mIL-5-anchored CCAR-T cells infusion, HDM-airway drip for allergic airway inflammation model, and sample analysis in BALB/c mice. i.v., intravenous administration. **b** Representative flow cytometry analysis of Eos proportion in BALF. BALF, bronchoalveolar lavage fluid. **c** Quantification of Eos proportion in BALF. Kruskal-Wallis test, ns, no significance, ^**^P < 0.01. **d** Cell count of Eos in BALF. Kruskal-Wallis test, ^**^P < 0.01^.^ **e** Histogram of Eos proportion in lung tissue. Kruskal-Wallis test, ^*^P < 0.05. **f** Representative flow cytometry analysis of Eos proportion in PB. PB, peripheral blood. **g** Quantification of Eos proportion in PB. One-way ANOVA corrected with the Tukey method, ^*^P < 0.05, ^**^P < 0.01. **h** Histogram of Eos proportion in BM. BM, bone marrow. Kruskal-Wallis test, ^*^P < 0.05, ^**^P < 0.01. **i** The concentration of IL-5 in BALF was determined by CBA kit. CBA, Cytometric Bead Array. Two-tailed Mann-Whitney test, ^**^P < 0.01. **j** Cell count of BALF total cells by microscope. One-way ANOVA corrected with the Tukey method, ^**^P < 0.01. **k** Representative images of the pulmonary sections stained with H&E. Scale bars, 100 μm. **l** Inflammation scores of the H&E-stained sections determined by semi-quantification. One-way ANOVA corrected with the Tukey method, ^*^P < 0.05, ^***^P < 0.001.

Next, we assessed the effect of CCAR-T cells in allergic airway inflammation mouse models^55–57^. In an acute asthmatic inflammation model, 3 × 10^6^ mIL-5-anchored CCAR-T cells were intravenously injected into recipient mice one week before administration of the extract of house dust mite (HDM) (Fig. 4a), and eosinophils were analyzed by flow cytometry using cell surface staining (Supplementary Fig. S7). Indeed, administration of mIL-5-anchored CCAR-T cells strikingly reduced both the proportion and the absolute number of eosinophils in bronchoalveolar lavage fluid (BALF) that are typically elevated during HDM-stimulated conditions (Fig. 4b–d). In addition, CCAR-T cells brought a significant decrease in eosinophil levels in lung tissue of HDM-treated mice (Fig. 4e), as well as in the peripheral blood (Fig. 4f, g) and bone marrow (Fig. 4h). Collectively, these data demonstrate that the mIL-5-anchored CCAR-T cells can efficiently target and eliminate eosinophils in the HDM-induced allergic airway inflammation model.

We next investigated the impact of mIL-5-anchored CCAR-T cells on the level of airway inflammation. IL-5 is a type 2 cytokine that promotes differentiation and activation of eosinophils and is, therefore, an essential biomarker for asthma^58,59^. We discovered that mIL-5-anchored CCAR-T cells resulted in a significant decrease in IL-5 level in BALF (Fig. 4i). Administration of CCAR-T cells also reduced the total number of inflammatory cells in BALF (Fig. 4j), indicating the remission of inflammatory infiltration in the airway. We further assessed the inflammation level in hematoxylin-eosin (H&E) stained pulmonary sections by semi-quantification. The notable differences in inflammatory scores following CCAR-T cells administration confirmed that mIL-5-anchored CCAR-T cells protect against airway inflammation (Fig. 4k, l).

### Long-term efficacy of CCAR-T cells

**Fig. 5:**
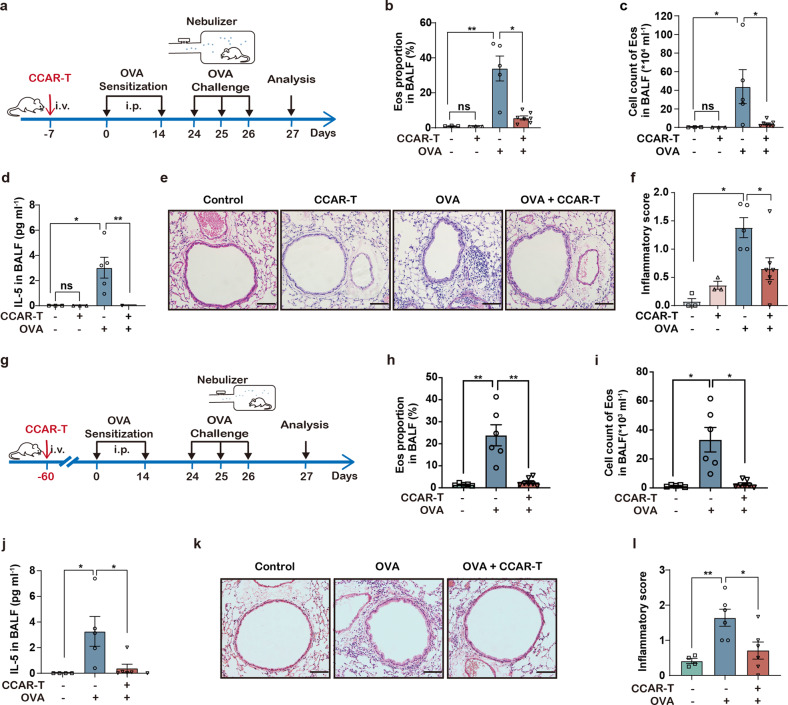
IL-5-anchored CCAR-T cells exhibit durable asthma control. **a** Timeline of intravenous injection of mIL-5-anchored CCAR-T cells, OVA-aerosol administration for the asthma model, and sample analysis in BALB/c mice. **b** Flow cytometry analysis of Eos proportion in BALF. ^*^P < 0.05, ^**^P < 0.01 by two-tailed Welch’s *t*-test. **c** Cell count of Eos in BALF in OVA-induced asthma model. ^*^P < 0.05 by two-tailed Mann-Whitney test. **d** The concentration of IL-5 cytokine in BALF was determined by CBA. ^*^P < 0.05, ^**^P < 0.01 by two-tailed Mann-Whitney test. **e** Representative images of the pulmonary sections stained with H&E. Scale bars, 100 μm. **f** Inflammation scores of the H&E-stained sections determined by semi-quantification. ^*^P < 0.05 by two-tailed Mann-Whitney test. **g** Timeline of CCAR-T cell administration and sample analysis in the allergic airway inflammation model. **h** Flow cytometry analysis of Eos proportion in BALF. ^**^P < 0.01 by two-tailed Mann-Whitney test. **i** Cell count of Eos in BALF. ^*^P < 0.05 by two-tailed Mann-Whitney test. **j** The secretion of IL-5 cytokine in BALF was determined by CBA. CBA, Cytometric Bead Array. Two-tailed Mann-Whitney test, ^*^P < 0.05. **k** Representative images of the pulmonary sections stained with H&E. Scale bars, 100 μm. **l** Inflammation scores of the H&E-stained sections determined by semi-quantification. ^*^P < 0.05, ^**^P < 0.01 by two-tailed Welch’s *t*-test.

To evaluate the duration of efficacy of the current IL-5-anchored CCAR-T strategy, we tested the effect of mIL-5-anchored CCAR-T cells in the ovalbumin (OVA)-containing aerosols inhalation induced airway inflammation model. In the one-month model (Fig. 5a), we observed mIL-5-anchored CCAR-T-dependent reduction in the eosinophil levels in BALF (Fig. 5b, c), lung tissue (Supplementary Fig. S8a), and peripheral blood (Supplementary Fig. S8b), and decreased IL-5 levels in BALF (Fig. 5d) as well as alleviated inflammation scores in lungs (Fig. 5e, f).

Moreover, the IL-5-anchored CCAR-T cells maintained effective control of asthma-related conditions for up to three months (Fig. 5g–l; Supplementary Fig. S9), including the eosinophil levels in BALF (Fig. 5h, i), peripheral blood (Supplementary Fig. S9a) and bone marrow (Supplementary Fig. S9b), IL-5 levels in BALF (Fig. 5j) as well as the inflammatory infiltration in the airway (Fig. 5k, l). These findings imply that the CCAR-T concept might set a new standard for long-term inflammation protection for asthmatic patients.

Additionally, no differences in the proportion of Th1 cells, Th2 cells, or Treg cells could be observed following CCAR-T transfer, excluding putative effects of CCAR-T cells on endogenous T cell responses (Supplementary Fig. S10). Also, no significant impact on systemic inflammatory biomarkers, serum IL-6 and IFN-γ, was observed (Supplementary Fig. S11).

## Discussion

Eosinophilic inflammation plays a prominent role in SEA. According to reports^60–62^, eosinophil depletion does not increase the risk of helminth infection or affect the vaccine responses. Although current management or add-on therapies for SEA can control the symptoms to a certain extent, inflammation relief is short-lasting, and asthma exacerbation continues. To address these concerns, we developed a cellular adoptive immunotherapy using design principles adopted from chimeric antigen receptor-T cells^29,30^ and applied it to allergic asthma. To avoid anaphylaxis reactions typically observed in scFv-dependent CAR-T therapies for cancer, we employed a ligand-anchored CAR design that allows cytokines to trigger target-specific T-cell killing. From a cell engineering perspective, the design of scFv-independent CARs is also highly advantageous in terms of time- and resource-efficiency, as the costly large-scale screening for antibody moieties can be omitted.

In this study, we have engineered the IL-5-anchored CCAR-T cells and verified their killing capacity in vitro and in mice. We showed that the IL-5-anchored CCAR-T cells exhibited efficacious and persistent control of eosinophilic asthma conditions in both the HDM and OVA-stimulated acute inflammatory asthma models. Furthermore, IL-5-anchored CCAR-T cells maintained a constant effect on eosinophil reduction, IL-5 reduction, and prevention of airway inflammation over three months, exceeding the typical active therapeutic window of single mAb-injections of 4 weeks^11^.

During traditional CAR-T treatment, the monoclonal antibodies-derived scFvs could induce immune responses due to their high immunogenicity^43^. In this case, anti-mIL-5Rα CAR carrying scFvs might elicit anti-CAR responses, especially in the hypersensitive immune environment of allergen-induced asthma models involved in this study, which might contribute to the treatment failure of anti-mIL-5Rα CAR-T cells in vivo.

When exposed to allergens, eosinophil progenitors rapidly differentiate into a large number of mature eosinophils. IL-5Rα is highly expressed on the surface of both eosinophil progenitors and mature eosinophils, while CCR3 is mainly expressed on mature eosinophils^63^. This might explain why CCAR-T cells targeting CCR3 failed to reduce eosinophils in vivo.

As the chronicity and the need for long-term or even life-long therapy are severe challenges during the treatment of eosinophilic diseases^64^, IL-5-anchored CCAR-T cells therapy is expected to solve these problems. Thus, the cytokine-anchored CCAR-T strategy not only showed unprecedented medical potential in SEA therapy but might also kick off a new era of cell-based precision medicine for the treatment of other eosinophilic diseases, such as chronic rhinosinusitis, eosinophilic esophagitis, and even chronic eosinophilic leukemia^64^.

## Materials and methods

### Antibodies

To detect the activation of the Jurkat cell line, we used anti-human CD69 PE (BioLegend). To analyze the phenotype of mouse eosinophils in BALF or lung tissues, we used anti-mouse CD45 PE-CY7 (BioLegend), anti-mouse SiglecF PE (BD), anti-mouse F4/80 APC-CY7 (BioLegend), anti-mouse CD11b FITC (BioLegend) and anti-mouse CD11c APC (BioLegend). To detect mIL-5Rα positive cells, we used anti-mouse CD125 AF488 (BD). To detect mIL-5-anchored CCAR and hIL-5-anchored CCAR, we used anti-mouse/human IL-5 PE (BioLegend) or anti-HA.11 Epitope Tag AF647 (BioLegend). To detect anti-hIL-5Rα CAR, hCCL11-anchored CCAR, and hCCL24-anchored CCAR, we used anti-HA.11 Epitope Tag AF647 (BioLegend).

### Animals and cell lines

Wild-type BALB/c mice were provided by the Animal Center of Slaccas (Shanghai, China). NCG (NOD/ShiLtJGpt-*Prkdc*^em26Cd52^*Il2rg*^em26Cd22^/Gpt) mice were provided by GemPharmatech Co., Ltd (Nanjing, China). All mouse experiments were performed under the stipulations approved by the Ethics Committee for Animal Studies of Zhejiang University (ZJU20210182). U2OS cells, Plat-E cells, and HEK293T cells were cultured in Dulbecco’s modified Eagle’s medium (DMEM) (HyClone) supplemented with 10% FBS (Gibco) and 100 IU /mL penicillin and streptomycin (10 mg/mL, Gibco). Jurkat cells were cultured in RPMI-1640 medium (HyClone) supplemented with 10% FBS (Gibco) and 100 IU/mL penicillin and streptomycin (10 mg/mL, Gibco).

### Isolation, expansion, and genetic modification of primary mouse T cells

Splenocytes were harvested from the BALB/c mice. Primary CD3^+^ T cells, which included CD4^+^ T cells and CD8^+^ T cells, were purified from the splenocytes using the mouse CD3 T cell isolation kit (BioLegend) and were cultured at 10^6^/mL in RPMI-1640 medium (HyClone) supplemented with 10% FBS (Gibco), HEPES (10 mM, Solarbio), sodium pyruvate (1 mM, GENOM), 1× non-essential amino acids (Gibco), β-mercaptoethanol (50 μM, Sigma) and 100 IU/mL penicillin and streptomycin (10 mg/mL, Gibco). T cells were stimulated under the condition of anti-mouse CD3 antibody (1 μg/mL, BioLegend) and anti-mouse CD28 antibody (2 μg/mL, BioLegend). 48 h after T cells stimulation, T cells were transduced with retroviral supernatants from the Plat-E cell line in the presence of polybrene (6 μg/mL, Yeasen) by centrifugal infection. T cells were analyzed by flow cytometry 2 days after transduction and were used for further experiments.

### Isolation, expansion, and genetic modification of human T cells

Peripheral blood was obtained from the healthy donors. Blood sampling was performed following the required ethical procedures. Lymphocytes were isolated by density gradient centrifugation following the manual of the Human Lymphocyte Separation Medium (DAKEWE). Human T cells were purified using the human CD3 T cell isolation kit (BioLegend), stimulated with CD3/CD28 T cell Activator Dynabeads (Gibco) and cultured at 10^6^/mL in X-VIVO 15 Serum-free Hematopoietic Cell Medium (Lonza), supplemented with 5 ng/mL human IL-7 (PeproTech) and 5 ng/mL human IL-15 (PeproTech)^40,65^, with slight modification. 48 h after T cell stimulation, T cells were transduced with lentiviral supernatants from 293 T cell line in the presence of polybrene (6 μg/mL, Yeasen) by centrifugal infection. T cells were analyzed by flow cytometry 4 days after transduction and were used for further experiments. All human subjects were informed and signed informed consent prior to inclusion in the study and all human cell isolation and related experiments were approved by the Ethics Committee for Human Studies of Second Affiliated Hospital of Zhejiang University School of Medicine (2019 NO.388).

### Cytotoxic activity assay

The cytotoxicity of CCAR-T cells, CAR-T cells, or UTD-T cells was determined by the luciferase-based assay as described previously^40,65^. In detail, 1 × 10^4^ target cells, stably expressing firefly luciferase through retrovirus infection, were cocultured with killing cells at the indicated T/target ratios in white 96-well plates (Costar) for indicated incubation time. Target cells alone were plated at the same cell density for determining the maximal luciferase expression (relative light units, RLU). The culture medium was discarded carefully and 15 μg D-luciferin (GoldBio) in 100 μL PBS was added to each well after coculture. Emitted light was detected by the luminescence plate reader (SynergyMx M5, Molecular Devices) and was converted into lysis (%) according to the previous report^40^ to characterize the cytotoxicity.

$${{{\mathrm{Lysis}}}}\left( {{{\mathrm{\% }}}} \right)\;{{{\mathrm{was}}}}\;{{{\mathrm{determined}}}}\;{{{\mathrm{as}}}}\left( {{{{\mathrm{1-RLUsample/RLUmax}}}}} \right){{{\mathrm{ \times 100}}}}{{{\mathrm{.}}}}$$

### Adhesion frequency assay

The preparation of the red blood cells (RBCs) and the experimental procedure of adhesion frequency assay have been described in detail previously^53,54^. Briefly, for the preparation of the hIL-5Rα-coated RBCs, the human IL-5Rα (hIL-5Rα) extracellular domain linked with AviTag was expressed, purified, and biotinylated. The biotinylated hIL-5Rα was linked to streptavidin-coated RBCs (SA-RBCs) to produce hIL-5Rα-coated RBCs which were then used for the adhesion frequency assay. For the adhesion frequency assay, it was used for measuring the in-situ binding kinetics of the hIL-5Rα and the anti-hIL-5Rα CAR/CCAR. In brief, this assay utilized micromanipulation to precisely operate the contact and retraction between the hIL-5Rα-coated RBCs and the anti-hIL-5Rα CAR/CCAR Jurkat cells.

The binding frequency *P*_a_ was acquired with definite contact area *A*_c_ and a series of preset contact time *t*_c_ through 50 contact-retraction cycles. And the in-situ effective binding affinity *A*_c_*K*_a_ and the off-rate *k*_off_ were then calculated by the probabilistic kinetic model:

$$P_{\mathrm{a}} = 1 - {\mathrm{exp}}(-m_{\mathrm{r}}m_{{\mathrm{hIL}}{\hbox{-}}5{\mathrm{R}}\alpha}A_{\mathrm{c}}K_{\mathrm{a}}(1 - {\mathrm{exp}}(k_{\mathrm{off}}))),$$

Where *m*_r_ and *m*_hIL-5Rα_ are respective CAR/CCAR and hIL-5Rα molecular densities, which are determined by standard calibration beads on flow cytometry. In-situ effective on-rate *A*_c_*k*_on_ was then calculated by: *A*_c_*k*_on_ = *A*_c_*K*_a_ × *k*_off_.

### Xenograft model in NCG mice

For the in vivo CCAR-induced Jurkat cell activation assay, the NCG mice were injected intraperitoneally with 1 × 10^7^ hIL-5Rα^+^ U2OS cells and 1 × 10^7^ hIL-5-anchored CCAR-Jurkat cells at 0 h. Mice were sacrificed at 3 h or 6 h, and the CCAR-Jurkat cells were harvested from intraperitoneal lavage fluids and analyzed by flow cytometry.

For the in vivo CCAR-induced T cell cytotoxicity assay, the NCG mice were injected intraperitoneally with 3 × 10^5^ hIL-5Rα^+^ U2OS cells expressing firefly luciferase and 1 × 10^6^ murine primary hIL-5-anchored CCAR-T cells or human primary hIL-5-anchored CCAR-T cells at day 0. 24 h later, the bioluminescence imaging of the mice was performed 10 min after intraperitoneal injection of 100 μL D-luciferin (30 mg/mL, GoldBio) on an IVIS Spectrum imaging system (Caliper) and the average radiance of hIL-5Rα^+^ U2OS cells was measured through the Living Image software (Caliper).

### Murine bone marrow-derived eosinophils (BMDE) differentiation in vitro

The isolation and culture of BMDE were performed as described previously^66,67^, with slight modification. Bone marrow cells were harvested from the tibias and femurs of BALB/c mice. The cells were cultured at 10^6^/mL in IMDM medium (Invitrogen) containing FBS (20%, Gibco), L-glutamine (2 mM), sodium pyruvate (1 mM, GENOM), 1× non-essential amino acids (Gibco), β-mercaptoethanol (50 μM, Sigma) and penicillin (100 IU/mL) and streptomycin (10 mg/mL, Gibco). MSCF (100 ng/mL, PeproTech) and mFLT3 ligand (100 ng/mL, PeproTech) were supplemented during the first 4 days. On day 4, the cells were washed and reseeded in the fresh IMDM medium supplemented with mIL-5 (10 ng/mL, Minneapolis) for the next 4 days. On day 8, the cells were harvested and analyzed by flow cytometry.

### Mouse model of eosinophilic asthma

#### OVA-induced asthma model

BALB/c mice were sensitized with 200 μL of 80 μg OVA (Sigma-Aldrich) emulsified in the aluminum adjuvant (Thermo Scientific) through intraperitoneal injection on day 0 and day 14, and control mice were administered with 200 μL saline (NS). On days 25-27, sensitized mice were challenged with 1.5% OVA in saline through aerosol administration for 40 min every time by an ultrasonic atomizer (Devilbiss). 24 h after the final challenge, mice were sacrificed for analysis.

#### HDM-induced asthma model

BALB/c mice received HDM (100 μg, D. pteronyssinus) in 50 μL saline through airway drip on day 0, day 7, and day 14, as described previously^56,68^. Control mice received 50 μL saline (NS) in the same way. Then mice were sacrificed 72 h after the final airway drip for analysis.

### Detection of the inflammatory factors

The concentration of IL-5 in BALF supernatants was measured by mouse IL-5 enhanced sensitivity cytometric bead array assay (Enhanced CBA, BD), serum IL-6 by mouse IL-6 Enhanced CBA (BD), serum IFN-γ by mouse IFN-γ Enhanced CBA (BD), serum IL-13 by mouse IL-13 Enhanced CBA (BD) and serum IL-4 by mouse IL-4 Enhanced CBA (BD) following the manufacturer’s manual. The human IFN-γ was measured by the human IFN-γ ELISA kit (AbClonal).

### Perivascular inflammation score

The pulmonary sections were embedded in paraffin and stained with hematoxylin-eosin (H&E) after fixation. The score of the perivascular inflammation was determined by the degree of inflammatory cell infiltration and was assessed as 0–3 on a subjective scale, as described previously^69,70^, with slight modification. Briefly, 0 means no or occasional inflammatory cells distributed in the perivascular space; 1 means 1 layer of inflammatory cells surrounded in the perivascular space; 2 for 2–5 layers of inflammatory cells; 3 for more than 5 layers of inflammatory cells.

### Statistical analysis

Data are presented as mean ± SEM. Statistical analyses were performed using GraphPad Prism software 8.0. Comparisons in each experiment were described in the figure legends. All representative data were replicated in at least three independent experiments.

## Supplementary information

Supplementary Figures

## Data Availability

The main data supporting the results in this study are available within the paper and its Supplementary Figures. The data generated and analyzed during the study are available from the corresponding author on reasonable request.
