# Supplementary material for: Treatment of allergic eosinophilic asthma through engineered IL-5-anchored chimeric antigen receptor T cells
Source: Cell Discov. 2022 Aug 16;8:80. doi: 10.1038/s41421-022-00433-y (PMC9381771; doi:10.1038/s41421-022-00433-y)
Supplement: Supplementary file 1 — Supplementary Figures [file 41421_2022_433_MOESM1_ESM.pdf]

Supplementary Information

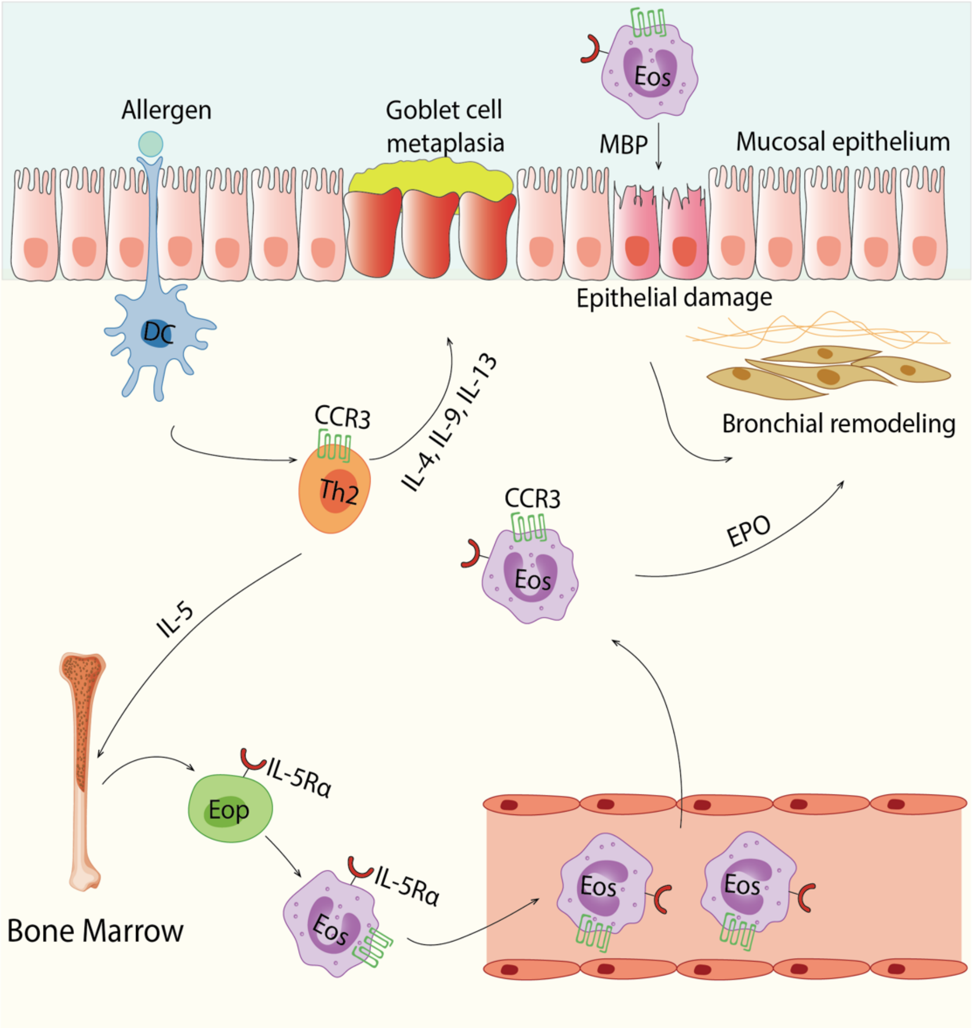

Supplementary information, Fig. S1. Schematic representation of the dominant cells with specific surface targets in asthma. Eop, eosinophil progenitor; DC, dendritic cell; EPO, eosinophil peroxidase; MBP, major basic protein.

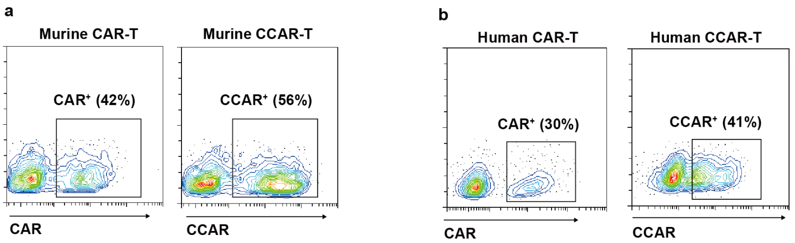

Supplementary information, Fig. S2. CAR/CCAR expression at the cell surface. a Representative flow cytometry analysis of the CAR or the CCAR surface expression on anti-hIL-5Ra CAR-T cells or the hIL-5-anchored CCAR-T cells (murine primary T cells) at 48 h post transduction. **b** Representative flow cytometry analysis of the CAR surface expression on anti-hIL-5Ra CAR-T cells and the CCAR surface expression on hIL-5-anchored CCAR-T cells from healthy human donors on day 6 post transduction.

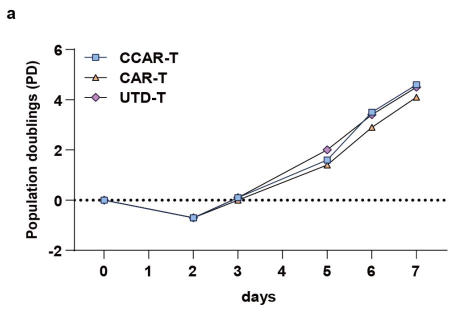

Supplementary information, Fig. S3. Population doublings of the CCAR-T cells from human donors. Population doublings of hIL-5-anchored CCAR-T cells, anti-hIL-5Rα CAR-T cells and UTD-T cells from healthy human donors.

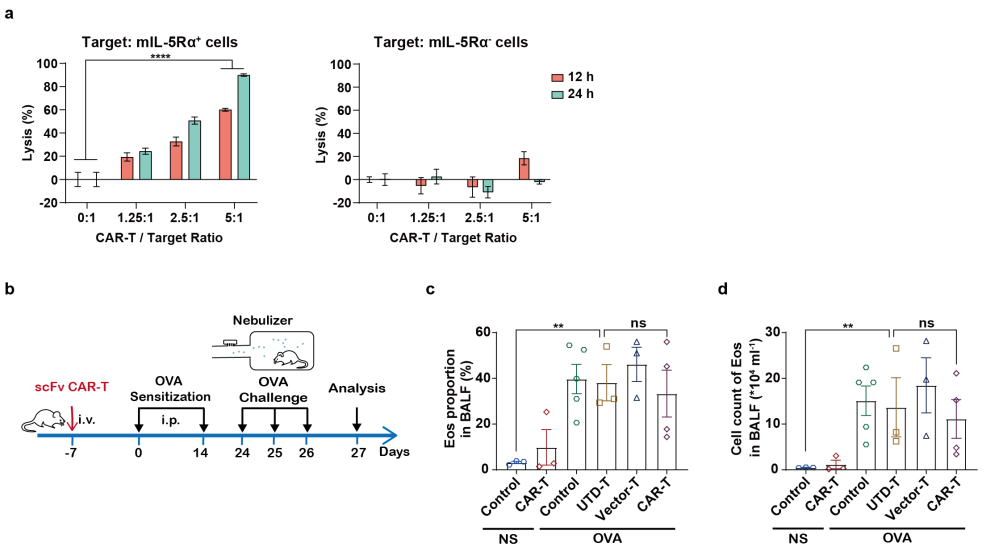

Supplementary information, Fig. S4. Efficacy of the anti-mIL-5Rα CAR-T cells in the asthma model. a Cytotoxic activity of anti-mIL-5Rα CAR-T cells as determined by a bioluminescence assay using luciferase-expressing mIL-5Rα$^+$ or mIL-5Rα$^-$ target cells. Primary T cells were isolated from BALB/c mice. Differences between the CAR-T cell-treated and control group were examined by the two-way ANOVA corrected with the Tukey method. $^{****}P < 0.0001$. **b** Timeline of intravenous injection of anti-mIL-5Rα CAR-T cells, OVA-aerosol administration for the asthma model, and sample analysis in BALB/c mice. **c** Flow cytometry analysis of Eos proportion in BALF in OVA-induced asthma model. UTD-T, un-transduced T cells; Vector-T, T cells transduced with a truncated CAR; CAR-T, anti-mIL5Ra scFv CAR-T cells. ns, no significance; $^{**}P <$ 0.01 by two-tailed t-test. **d** Cell count of Eos in BALF in OVA-induced asthma model. ns, no significance; $^{**}P < 0.01$ by two-tailed t-test.

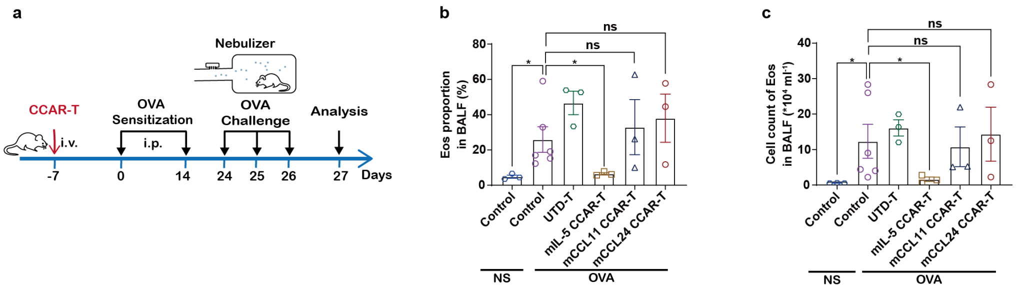

Supplementary information, Fig. S5. Comparison of the IL-5-anchored, CCL11-anchored and CCL24-anchored CCAR-T cells in the asthma model. a Timeline of intravenous injection of mIL-5-anchored, mCCL11-anchored, or mCCL24-anchored CCAR-T cells, OVA-aerosol administration for the asthma model, and sample analysis in BALB/c mice. **b** Flow cytometry analysis showing Eos proportion in BALF in OVA-induced asthma model. ns, no significance; $^{*}P < 0.05$ by two-tailed Mann-Whitney test. UTD-T, un-transduced T cells. **c** Cell count of Eos in BALF in OVA-induced asthma model. ns, no significance; $^{*}P < 0.05$ by two-tailed Mann-Whitney test.

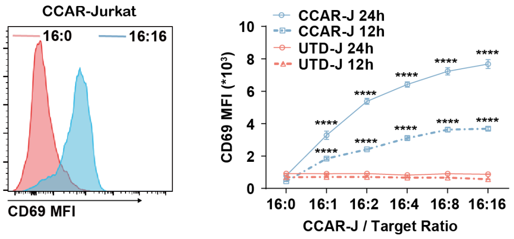

Supplementary information, Fig. S6. The activation of the hIL-5-anchored CCAR-Jurkat cells in vitro. Jurkat cells were transduced with hIL-5-anchor CCAR comprising a human IL-5 linked to human CD28 costimulatory and CD3ζ signalling domains (h.IL5-h.28z). Flow cytometry analysis of CD69 surface expression on hIL-5-anchor CCAR-Jurkat cells after coculture with hIL-5Rα^{+} target cells at indicated CCAR-T to target ratios and different time points. $^{****}P < 0.0001$ by two-way ANOVA comparing CCAR-Jurkat to UTD-Jurkat cells at each time point corrected with the Tukey method.

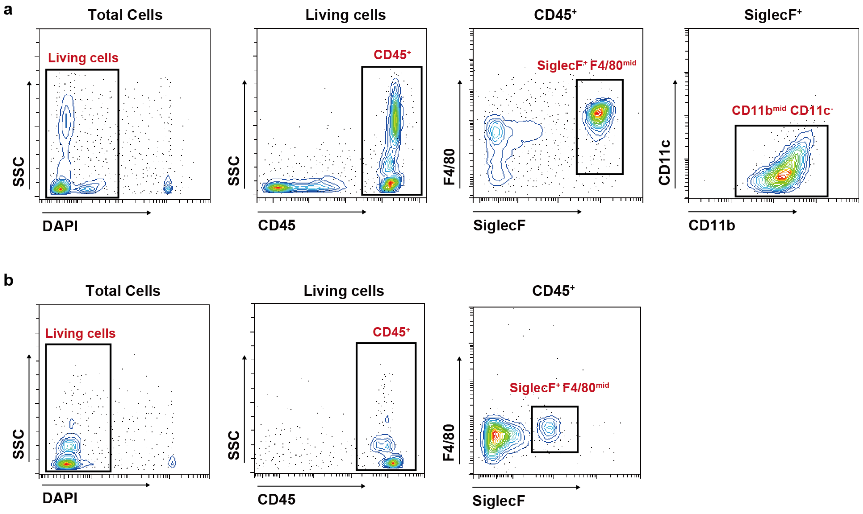

Supplementary information, Fig. S7. Flow cytometric analysis of the mouse eosinophils. a The gating strategy of Eos in BALF or lung tissue. **b** The gating strategy of Eos in PB or BM. PB, peripheral blood. BM, bone marrow.

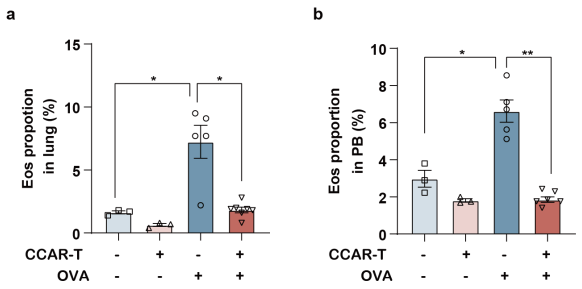

Supplementary information, Fig. S8. CCAR-T cells reduced the eosinophil levels in vivo over one month. a Flow cytometry analysis of Eos proportion in the lung in the OVA-induced asthma model over one month. Two-tailed Welch's t-test, $^{*}P < 0.05$. **b** Flow cytometry analysis of Eos proportion in PB in the OVA-induced asthma model over one month. PB, peripheral blood. Two-tailed Welch's t-test, $^{*}P < 0.05$, $^{**}P < 0.01$.

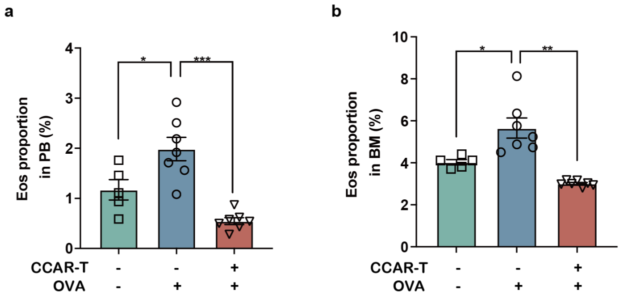

Supplementary information, Fig. S9. CCAR-T cells reduced the eosinophil levels in vivo over three months. a Flow cytometry analysis of Eos proportion in PB in the OVA-induced asthma model over three months. PB, peripheral blood. Two-tailed t-test, $^{*}P < 0.05$, $^{***}P < 0.001$. **b** Flow cytometry analysis of Eos proportion in BM in the OVA-induced asthma model over three months. BM, bone marrow. Two-tailed Welch's t-test, $^{*}P < 0.05$, $^{**}P < 0.01$.

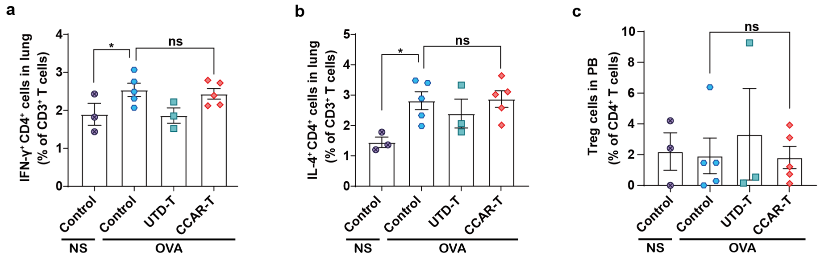

Supplementary information, Fig. S10. Endogenous T cell responses. a Flow cytometry analysis showing the proportion of Th1 cells (IFN-γ^+ CD4$^+$ cells) in the lung. **b** Flow cytometry showing the proportion of Th2 cells (IL-4$^+$ CD4$^+$ cells) in the lung. **c** Flow cytometry showing the proportion of Treg cells (CD4$^+$ CD25$^+$ FOXP3$^+$ cells) in PB. PB, peripheral blood.

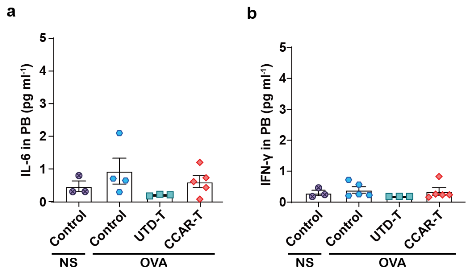

Supplementary information, Fig. S11. Serum cytokine levels after CCAR-T cell infusion. a, b Serum IL-6 (**a**) and IFN-γ (**b**) in the OVA-induced asthma model were determined by the CBA kit.
